# Association of oncogene mutations with clinical and histopathological characteristics in patients with metastatic melanoma^☆^

**DOI:** 10.1016/j.abd.2025.501260

**Published:** 2026-01-07

**Authors:** Julia Amaral Quintiliano, Adauto Ferreira Nunes, Luiza Pinheiro-Hubinger-Stauffer

**Affiliations:** a Pathology Department, Instituto Lauro de Souza Lima, Bauru, SP, Brazil; b Pathology Department, Hospital Amaral Carvalho, Jau, SP, Brazil

Dear Editor,

Melanoma is recognized as a highly aggressive form of skin cancer and exhibits the highest mutation rates of all solid tumors. Among the genetic alterations identified, mutations in specific oncogenes, particularly in the BRAF gene, are the most prevalent.^1^ Characterizing the genetic profile of tumors is essential, as several approved and investigational therapies are effective only in the presence of specific mutagenic alterations.^2^

The clinical and epidemiological investigation of factors associated with metastatic melanoma can offer valuable insights into disease progression and inform preventive strategies and targeted therapeutic approaches. In this context, we retrospectively evaluated the mutational status of the BRAF, PDGFRA, and C-KIT genes in patients with metastatic melanoma treated at a Brazilian tertiary oncology hospital, correlating these findings with epidemiological and clinical parameters.

Medical records of 94 patients diagnosed with melanoma and treated between 2015 and 2022 at Hospital Amaral Carvalho (HAC), Jaú, São Paulo, Brazil, were reviewed. Clinical and histopathological data were collected. Exon 15 of the BRAF gene (codon 600 and adjacent regions) was sequenced in all cases. In selected samples, additional analyses for C-KIT and PDGFRA mutations were performed based on physician requests.

DNA was extracted using the QIAAMP DNA FFPE Tissue Kit (QIAGEN). PCR amplification employed primers previously described by Qiu et al.^3^ and Braggio et al.^4^ DNA sequencing was conducted on an ABI PRISM 3100 Genetic Analyzer-3130xl system.

Descriptive and inferential statistical analyses were performed using Pearson’s chi-square test or Fisher’s exact test, binomial logistic regression (variables included age, gender, patient status, primary tumor site, histological subtype, ulceration, Breslow thickness, and mitotic index), and Kaplan-Meier survival analysis. Cases with missing data were excluded from specific analyses. A significance level of 5% was adopted. This study was approved by the Ethics Committees of the Lauro de Souza Lima Institute and Hospital Amaral Carvalho (Protocol 58842922.0.3001.5434).

Table 1 summarizes the demographic and tumor-specific characteristics of the cohort. The most frequent site of metastasis was the regional lymph nodes (61.7%), followed by the lungs (13.8%), bones (6.4%), liver (4.3%), and other locations (13.8%) (Supplementary Table S1).

**Table 1:** Clinical characteristics of the patients and anatomopathological variables of the primary tumor.

Variable	Category	n	%
Gender	Female	45	47.9
	Male	49	52.1
Age at diagnosis	<40 years old	17	18.1
	40‒59 years old	37	39.4
	>60 years old	40	42.5
Primary tumor site	Head or neck	18	19.1
	Upper limb	09	9.6
	Trunk	26	27.7
	Lower limb	33	35.1
	Unidentified	08	8.5
Histological subtype	Superficial extensive	29	30.9
	Nodular	26	27.6
	Acral	11	11.7
	Lentigo maligno	01	1.1
	Others	03	3.2
	No information	24	25.5
Breslow index	<1 mm	13	13.8
	1.01 – 2 mm	06	6.4
	2.01 – 4 mm	22	23.4
	>4 mm	31	33.0
	No information	22	23.4
Ulceration	Yes	44	46.8
	No	50	53.2
Mitotic index	<1 mitosis/mm^2^	15	15.9
	>1 mitosis/mm^2^	45	47.9
	No information	34	36.2
LDH at diagnosis	Low	13	13.8
	Normal	41	43.6
	High	10	10.6
	No information	30	32.0
LDH at metastasis	Low	05	5.3
	Normal	29	30.9
	High	17	18.1
	No information	43	45.7
Patient Status	Alive	32	34.0
	Death	62	66.0
Overall survival	<5-years	45	72.6
	>5-years	17	27.4
Observed survival	>5-years	24	75.0
	<5-years after diagnosis	8	25.0

Among the 94 patients, 41 (43.6%) exhibited wild-type BRAF (BRAF-WT) and 53 (56.4%) harbored BRAF mutations: 48 (51%) had the V600E mutation, 4 (4.3%) had the V600 K mutation, and 1 (1.1%) had the L597Q mutation.

Nineteen patients underwent analysis for C-KIT mutations: 14 (73.7%) were wild-type, and 5 (26.3%) presented mutations (two in exon 17, and one each in exons 13, 11, and 9). Among five acral melanoma biopsies analyzed for C-KIT, 2 (40%) harbored mutations (in exons 9 and 13). The remaining C-KIT–mutated tumors were classified as nodular, polypoid-nodular, or superficial extensive melanomas.

Twelve patients were analyzed for PDGFRA mutations, all of whom were wild-type.

The association between BRAF mutational status and clinical-pathological variables is presented in Table 2. BRAF mutations were significantly associated with younger age at diagnosis (p = 0.0085).

**Table 2:** Relationship between clinical-pathological variables and the presence of BRAF mutation.

Variable	Category	BRAF-MUT	BRAF-WT	Total	p-value^a,b^	Logistic Regression	
						OR (95% CI)	p-value
Gender	Female	26	19	45			
	Male	27	22	49	0.7938^b^		
Age at diagnosis, years	<40	14	03	17		0.946 (0.910–0.983)	0.004
	40‒59	23	14	37	**0.0085**^b^		
	>60	16	24	40			
Primary tumor site	Head or neck	11	07	18			
	Upper limbs	04	05	09			
	Trunk	17	09	26	0.3891^b^		
	Lower limbs	15	18	33			
Histological type	Superficial extensive	19	10	29		0.161 (0.0276–0.935)^d^	0.042^d^
	Others	18	23	41	0.074^b^		
Breslow index	<1 mm	09	04	13		2.490 (0.964–6.432)	0.060
	>1 mm	32	27	59	0.3709^a^		
Ulceration	Yes	22	22	44			
	No	26	15	41	0.2126^b^		
Mitotic index, mitosis/mm^2^	<1	08	04	12			
	>1	22	26	48	0.3334^a^		
LDH at diagnosis, U/L	Up to 480	30	24	54			
	Above 480	06	04	10	>0.9999^a^		
LDH at metastasis, U/L	Up to 480	19	15	24			
	Above 480	11	06	17	0.5461^b^		
cSurvival	<5-years	24	21	45			
	>5-years	26	15	41	0.3439^b^		

a Fisher analysis.

b Chi-Square analysis.

c Three patients with BRAF mutation and five BRAF-WT patients who were diagnosed with the disease less than 5-years ago and are still being monitored were excluded.

d Significance was found in univariate analysis for the acral subtype and BRAF-WT.

In univariate logistic regression (n = 56), younger age was associated with BRAF mutation (p = 0.004, OR = 0.946, 95% CI: 0.910–0.983). The acral subtype was associated with wild-type tumors and superficial extensive cases with the BRAF mutation (p = 0.042, OR = 0.161, 95% CI: 0.0276–0.935). Breslow thickness was marginally related to WT tumors (p = 0.060, OR = 2.490, 95% CI: 0.964–6.432). In multivariate analysis, younger age remained the only independent factor associated with BRAF mutation (p = 0.003). No statistically significant associations were observed for C-KIT mutations (Table 3).

**Table 3:** Relationship between clinical-pathological variables and presence of c-KIT mutation.

Variable	Category	KIT-MUT	KIT-WT	Total	p-value
Gender	Female	04	06	10	
	Male	01	08	09	0.3034^a^
Age at diagnosis	<60-years-old	00	06	06	
	>60-years-old	05	08	13	0.1280^a^
Primary tumor site	Head/neck/trunk	02	04	06	
	Upper and lower limbs	03	10	13	>0.9999^a^
Histological type	Superficial extensive	01	04	05	
	Others	04	06	10	0.6004^a^
Breslow index	<1 mm	01	02	03	
	>1 mm	04	07	11	>0.9999^a^
Ulceration	Yes	05	09	14	
	No	00	04	04	0.2778^a^
Mitotic index	<1 mitosis/mm^2^	00	02	02	
	>1 mitosis/mm^2^	05	08	13	0.5238^a^
LDH at diagnosis	Up to 480 U/L	04	06	10	
	Above 480 U/L	01	00	01	0.4545^a^
LDH at metastasis	Up to 480 U/L	01	06	07	
	Above 480 U/L	00	02	02	>0.9999^a^
*Survival	<5-years	03	06	09	
	>5-years	02	07	09	>0.9999^a^

* One KIT-WT patient who was diagnosed with the disease less than 5-years ago and is still being monitored was excluded.

a Fisher analysis.

At the time of data collection, the median survival time was 62-months for patients with BRAF-mutated tumors and 50-months for BRAF-WT patients. The Hazard Ratio (HR) for death in the BRAF-WT group was 1.331. For KIT-mutated patients, the median survival was 38-months, compared to 70-months in KIT-WT patients (HR = 1.145) (Fig. 1).

**Fig. 1:**
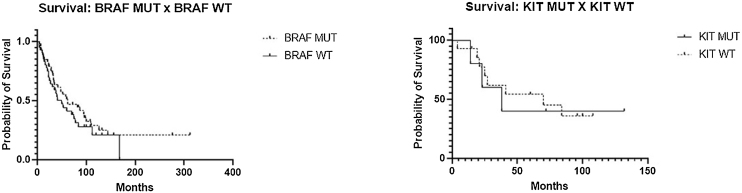
Survival curve BRAF-MUT × BRAF-WT and c-KIT-MUT × c-KIT-WT.

A slight male predominance was observed, and the onset of melanoma was predominantly from the fifth decade of life onwards, consistent with previous reports.^5^ Although the lower limbs were the most frequently affected primary site in this cohort, prior studies have more commonly reported the trunk and lower limbs.^5^ This discrepancy may reflect cultural or behavioral differences in sun exposure patterns.

Survival analysis revealed that 72.6% of deceased patients (n = 62) had a survival time of less than 5-years, while 75% of survivors (n = 32) remained alive beyond five years. Prognostic factors influencing melanoma survival include tumor thickness, ulceration, mitotic index, and metastatic burden.^6^

The most prevalent histological subtype was superficial extensive melanoma (30.9%), followed by the nodular subtype (27.6%). These findings may be attributable to the association between these subtypes and chronic or intermittent sun exposure. Additionally, more than 60% of patients exhibited a high Breslow index, 50% had a mitotic rate >1 mm^2^, and approximately one-third presented with tumors >4 mm in thickness ‒ all features linked to poor prognosis.^6^

Younger age was significantly correlated with BRAF mutation, in line with previous studies.^7^ The superficial extensive and nodular subtypes also demonstrated a higher frequency of BRAF mutations. Although not statistically significant, primary tumors located on the trunk and lower limbs tended to exhibit more BRAF mutations than those on the upper limbs or head and neck.

While the majority of patients with lower Breslow thickness and lower mitotic index harbored BRAF mutations, the prognostic relevance of BRAF status remains controversial, as some studies report no consistent correlation between BRAF mutations and histopathological parameters.^8^

KIT mutations were more frequent in female patients aged over 60-years. All KIT-mutated tumors exhibited ulceration and a mitotic index >1 mm^2^, both unfavorable prognostic markers.^6^ Notably, KIT mutations were identified in 40% of acral melanoma cases, a subtype typically classified as “non-solar” melanoma, potentially associated with mechanical trauma rather than UV radiation. Acral melanomas generally demonstrate a lower frequency of point mutations but a higher rate of gene copy number variations.^9^

The median 5-year survival was higher in patients with BRAF mutations and slightly lower in those without mutations. Reports indicate that patients with BRAF mutations treated with selective BRAF inhibitors can achieve a 5-year survival rate of up to 60% in some cases.^10^

The median 5-year survival was higher in patients without c-KIT mutations. Of the 19 patients tested for KIT mutations in this study, only 5 (26.3%) had mutations in this gene. Previous studies have reported that less than 10% of melanoma cases involve KIT mutations.^11^ Treatment with KIT inhibitors typically demonstrates lower therapeutic efficacy compared to other selective inhibitors, which may be associated with decreased survival.^2^

In this study, BRAF mutations were found to be associated with younger age and the superficial spreading subtype. No significant correlation was observed between KIT mutations and the examined variables. Our findings provide important insights into the clinical, pathological, and molecular characteristics of melanoma, offering valuable contributions to the development of future research focused on preventive and therapeutic strategies.

## ORCID ID

Quintiliano JA: 0009-0003-9488-1880

Nunes AF: 0000-0003-3473-9252

Pinheiro-Hubinger-Stauffer L: 0000-0001-7377-7652

## Financial support

This research did not receive any specific grant from funding agencies in the public, comercial, or not-for-profit sectors.

## Authors' contributions

Julia Amaral Quintiliano: Data collection, analysis and interpretation; statistical analysis; article writing.

Adauto Ferreira Nunes: Data interpretation; critical review of important intellectual content.

Luiza Pinheiro-Hubinger-Stauffer: Study conception and design; collection, analysis and interpretation of data; statistical analysis; research guidance; approval of the final version of the manuscript.

## Research data availability

The entire dataset supporting the results of this study was published in this article.

## Conflicts of interest

None declared.
