# Supplementary material for: Association of oncogene mutations with clinical and histopathological characteristics in patients with metastatic melanoma
Source: An Bras Dermatol. 2026 Jan 7;101(1):501260. doi: 10.1016/j.abd.2025.501260 (PMC12809065; doi:10.1016/j.abd.2025.501260)
Supplement: Supplementary file 1 [file mmc1.docx]

**ABD-D-25-00125_Supplementary Material**

**Supplementary Table S1** Distribution of metastases according to location.

| **Site** | **1^st^ Metastasis** | **2^nd^ Metastasis** | **3^rd^ Metastasis** | **4^th^ Metastasis** | **5^th^ Metastasis** |
| --- | --- | --- | --- | --- | --- |
| Regional lymph node | 58 (61.7%) | 18 (19.2%) | 02 (2.1%) | 00 | 00 |
| Skin | 02 (2.1%) | 06 (6.4%) | 04 (4.3%) | 03 (3.2%) | 00 |
| Liver | 04 (4.3%) | 10 (10.6%) | 04 (4.3%) | 08 (8.5%) | 01 (1.1%) |
| Lungs | 13 (13.8%) | 13 (13.8%) | 13 (13.8%) | 04 (4.3%) | 02 (2.1%) |
| Bones | 06 (6.4%) | 02 (2.1%) | 06 (6.4%) | 03 (3.2%) | 01 (1.1%) |
| Brain | 02 (2.1%) | 07 (7.5%) | 06 (6.4%) | 01 (1.1%) | 03 (3.2%) |
| Retroperitoneum | 01 (1.1%) | 04 (4.3%) | 09 (9.6%) | 03 (3.2%) | 03 (3.2%) |
| Others | 08 (8.5%) | 10 (10.6%) | 06 (6.4%) | 04 (4.3%) | 01 (1.1%) |
| No metastasis | 00 | 24 (25.5%) | 44 (46.8%) | 68 (72.3%) | 83 (88.3%) |
